# FBXW7 and the Hallmarks of Cancer: Underlying Mechanisms and Prospective Strategies

**DOI:** 10.3389/fonc.2022.880077

**Published:** 2022-04-19

**Authors:** Wenyue Shen, Quanwei Zhou, Chenxi Peng, Jiaheng Li, Qizhi Yuan, Hecheng Zhu, Ming Zhao, Xingjun Jiang, Weidong Liu, Caiping Ren

**Affiliations:** ^1^ Department of Neurosurgery, Xiangya Hospital, Central South University, Changsha, China; ^2^ National Clinical Research Center for Geriatric Disorders, Xiangya Hospital, Central South University, Changsha, China; ^3^ Cancer Research Institute, School of Basic Medical Science, Central South University, Changsha, China; ^4^ Changsha Kexin Cancer Hospital, Changsha, China; ^5^ The Key Laboratory of Carcinogenesis of the Chinese Ministry of Health and the Key Laboratory of Carcinogenesis and Cancer Invasion of the Chinese Ministry of Education, School of Basic Medicine, Central South University, Changsha, China

**Keywords:** FBXW7, hallmarks of cancer, metabolic reprogramming, therapeutic resistance, c-MYC, mTOR

## Abstract

FBXW7, a member of the F-box protein family within the ubiquitin–proteasome system, performs an indispensable role in orchestrating cellular processes through ubiquitination and degradation of its substrates, such as c-MYC, mTOR, MCL-1, Notch, and cyclin E. Mainly functioning as a tumor suppressor, inactivation of FBXW7 induces the aberrations of its downstream pathway, resulting in the occurrence of diseases especially tumorigenesis. Here, we decipher the relationship between FBXW7 and the hallmarks of cancer and discuss the underlying mechanisms. Considering the interplay of cancer hallmarks, we propose several prospective strategies for circumventing the deficits of therapeutic resistance and complete cure of cancer patients.

## 1 Introduction

Over the past few decades, accumulated knowledge in cancer research has gradually sketched the outline of cancer hallmarks in reference to molecular, biochemical, and cellular characteristics. In 2000, Hanahan and Weinberg generalized the hallmarks of cancers into 6 major biological capabilities, which comprises self-sufficiency in growth signals, limitless replicative potential, evading apoptosis, insensitivity to anti-growth signals, sustained angiogenesis, and invasion and metastasis (1). A decade later, this notion was further refined by introducing two emerging hallmarks: deregulating cellular energetics and avoiding immune destruction. The aforementioned eight hallmarks can be explained by two enabling characteristics: genome instability and mutation as well as tumor-promoting inflammation (2). Recently, this concept of cancer hallmarks has been updated again owing to the in-depth mining of cancer mechanisms, incorporating novel emerging hallmarks as well as enabling characteristics including unlocking phenotypic plasticity, non-mutational epigenetic reprogramming, polymorphic microbiomes, and senescent cells (3).

Rapid advances have gradually deepened our understanding of cancer biological behaviors. During these years, unremitting efforts in the fields of cancer biology have also shed light on its link with the main degradation mechanism of eukaryotes, that is, the ubiquitin–proteasome system (UPS). Through the degradation of proteins involved in a broad spectrum of cellular processes, such as cell cycle progression, signal transduction, and endocytosis, UPS is of great significance to regulate immune response, development, and apoptosis to maintain homeostasis, the abnormalities of which contribute to uncontrolled development, genomic instability, and even transformation of malignancy. The UPS-based degradation, termed ubiquitination, is a multi-level cascade associated with the covalent binding of ubiquitin throughout the course, mainly consisting of the following components: a ubiquitin-activating enzyme (E1), a ubiquitin-conjugating enzyme (E2), a ubiquitin ligase (E3), and a 26S proteasome. The initiation of ubiquitin-mediated degradation involves sequential reactions. After the ATP-dependent activation triggered by E1, the 76-amino-acid-residue protein ubiquitin (Ub) binds to E1 in a thiolester linkage. Then, Ub is transferred to E2, and subsequently transferred through E3 to substrate proteins (4, 5). Afterwards, a polyubiquitin chain can be formed for further degradation by the 26S proteasome complex.

The S-phase kinase-associated protein 1 (SKP1)-cullin-1 (CUL1)-F-box-protein (SCF) complex belongs to the really interesting new gene (RING) family of E3 ubiquitin ligases, mediating the degradation of ~20% proteins related to cell-cycle regulation, transcription, oncogenesis, and tumor suppression (6). The SCF complex contains the invariable subunits CULl, RING-box 1 (RBX1), and SKP1, as well as the variable component F-box proteins (7), which is characterized by an approximately 40-amino-acid motif and determines the substrate specificity of the SCF complex.

**Figure 1:**
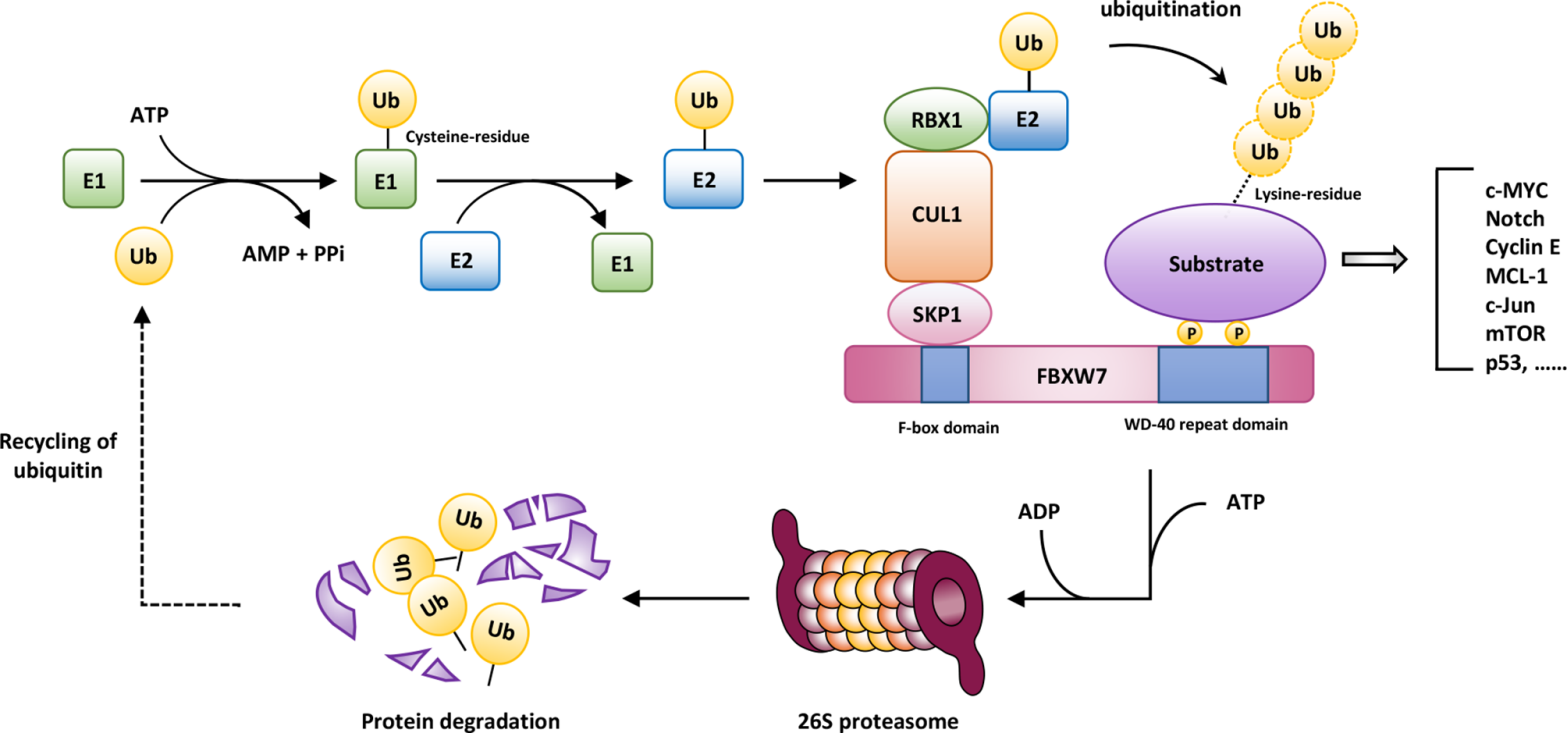
The ubiquitin-mediated degradation involved in FBXW7. After the ATP-dependent activation triggered by the ubiquitin-activating enzyme (E1), the 76-amino-acid-residue protein ubiquitin binds to E1 in a thiolester linkage. Then, ubiquitin (Ub) is transferred to the ubiquitin-conjugating enzyme (E2), and subsequently transferred through the ubiquitin ligase (E3) to substrate proteins. Afterwards, a polyubiquitin chain can be formed for further degradation by 26S proteasome complex, conferring the recycling of ubiquitin. AMP, adenosine monophosphate; ATP, adenosine triphosphate; CUL1, cullin 1; RBX1, RING-box 1; SKP1, S-phase kinase-associated protein 1.

F-box and WD repeat domain containing 7 (FBXW7, also known as AGO, hCdc4, FBW6, FBW7, SEL10 or FBX30) is a member of F-box proteins encoded by FBXW7 gene. The FBXW7 gene (Gene ID: 55294) maps to chromosome region 4q31.3 including 18 exons, where mutations are frequently detected in multiple human cancers, such as colorectal cancer (CRC) (8, 9), ovarian cancer (10), breast cancer (BC) (11), endometrial cancer (12), and human T-cell acute lymphoblastic leukemia (T-ALL) (13). Based on the statistics of COSMIC Cancer Gene Census list (Tier 1), the calculated population-level mutation proportion of FBXW7 is approximately 4.17% in Americans, ranking 28th among all genes counted (14). FBXW7 is composed of a tandem WD40-repeat domain, an F-box domain, a 5-residue tail, and an α-helical linker domain (15). Additionally, FBXW7 can be subdivided into three isoforms: FBXW7α localized in the nucleoplasm, FBXW7β residing in the cytoplasm, and FBXW7γ within the nucleolus (16), all of which recognize substrates through a conserved CDC4 phosphodegron (CPD) motif, requiring phosphorylation of substrates at particular sites by protein kinases such as the glycogen synthase kinase-3 (GSK3) to accelerate the ubiquitination and proteolysis process of substrates (17). As a consequence, the process of UPS-mediated proteolysis involved in FBXW7 is shown in **Figure 1**.

The mRNA and protein expression of FBXW7 can be detected almost in all human normal organs and tissues (18). FBXW7 protein expression level can be high, seen in lung, stomach, colon, kidney, breast, skin, etc.; it also can be medium, detected in rectum, liver, endometrium, cervix, etc. In addition, FBXW7 mRNA expression has organ specificity, such as brain (18). However, in malignant organs and tissues, FBXW7 protein expression is decreased in glioma, lung cancer, liver cancer, urothelial cancer, ovarian cancer, melanoma, etc. (18), and the expression levels of aforementioned cancers are positively correlated with prognosis in the majority (besides liver cancer and melanoma).

**Figure 2:**
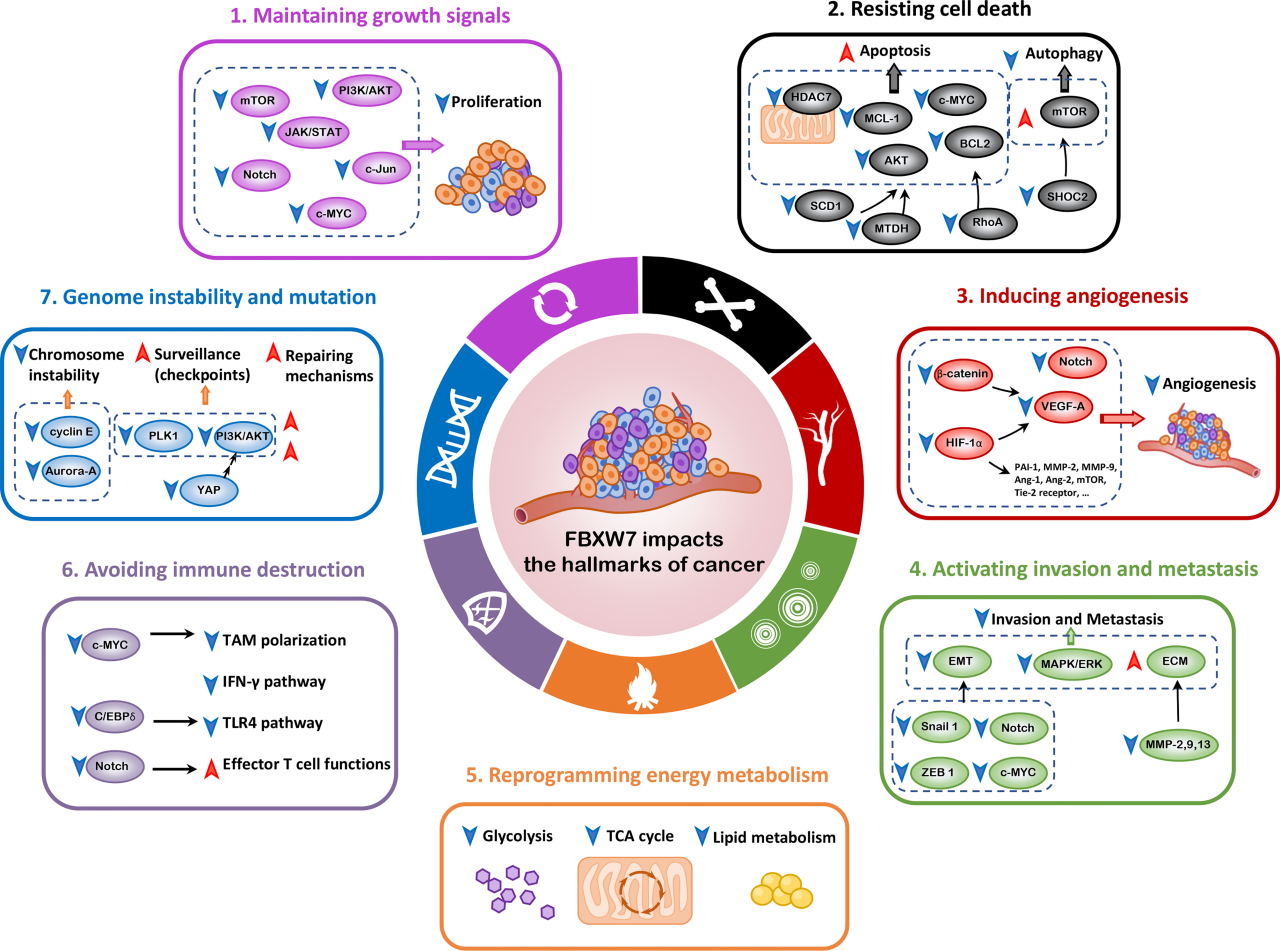
FBXW7 and the hallmarks of cancer. In this review, we mainly elaborate on seven hallmarks of cancer influenced by FBXW7, including maintaining growth signals, resisting cell death, inducing angiogenesis, activating invasion and metastasis, reprogramming energy metabolism, avoiding immune destruction, and genome instability and mutation. Ang-1, Angioprotein-1; BCL2, B cell lymphoma 2; C/EBPδ, CCAAT/enhancer binding protein δ; ECM, extracellular matrix; EMT, epithelial–mesenchymal transition; HDAC7, histone deacetylase 7; HIF-1α, hypoxia-inducible factor-1α; ICL, interstrand cross-link; MAPK/ERK, mitogen-activated protein kinase/extracellular signal–regulated kinase; MCL-1, myeloid cell leukemia-1; MMP, matrix metalloproteinase; MTDH, metadherin; NHEJ, nonhomologous end-joining; PAI-1, plasminogen activator inhibitor-1; PLK1, polo-like kinase 1; SCD1, stearoyl-CoA desaturase 1; VEGF-A, vascular endothelial growth factor-A; YAP, Yes-associated protein; ZEB1, zinc-finger E-box-binding homeobox 1.

It is universally acknowledged that FBXW7 exerts its major function as a tumor suppressor (19–21), orchestrating cellular processes by virtue of interacting with its substrates, such as c-MYC (22), Notch (23), cyclin E (11), myeloid cell leukemia-1 (MCL-1) (24), c-Jun (25), p53 (15, 26), and mechanistic target of rapamycin (mTOR) (27). As a consequence, in the following sections, we will mainly elaborate on the relationship between FBXW7 and cancer hallmarks and unravel the underlying mechanisms (**Figure 2**). In light of the interplay of cancer hallmarks, we highlight the prospects to battle against therapeutic resistance, one of the thorniest obstacles in the course of completely curing the patients.

## 2 FBXW7 and the Hallmarks of Cancer

### 2.1 Maintaining Growth Signals

Tumor cells are independent of exogenous growth signal stimulation and generate their own proliferative signals, which serves as one of the six hallmarks of cancer. FBXW7 reshapes the proliferative niches of tumor cells through ubiquitinating and degrading several crucial signal molecules, such as c-MYC, c-Jun, phosphatidylinositol 3-kinase (PI3K)/AKT, mTOR, Notch, as well as JAK/STAT signaling pathway.

c-MYC is a critical transcription factor in control of various biological processes such as proliferation, differentiation, and apoptosis (28, 29). Accumulated evidence has indicated that FBXW7 strictly regulates c-MYC turnover at the post-translational level following phosphorylation of its specific sites, where the sequential steps is associated with feedback mechanisms (30, 31). In adult T-cell leukemia/lymphoma (ATLL) cell lines, the mRNA and protein level of c-MYC is higher than normal due to the aberrations of FBXW7 expression, which is correlated with ATLL proliferation and poor prognosis of patients (32). Another study has disclosed that the oncogene Ecotropic viral integration site 5 (Evi5) accelerates laryngeal cancer cell proliferation by counteracting FBXW7, thus facilitating the accumulation of its substrate c-MYC (33).

c-Jun, a crucial member of the AP-1 family, participates in cellular proliferation, apoptosis, survival, and tumorigenesis. FBXW7 exerts its role as a component of c-Jun’s E3 ligase, and knockdown of FBXW7 confers the accumulation of c-Jun (25). In colon cancer cells, the lysine demethylase KDM5c could attenuate FBXW7-mediated degradation of c-Jun by epigenetically modifying FBXW7 and decreasing FBXW7 transcription, further promoting cell proliferation (34).

The PI3K/AKT signaling pathway regulates the functions of cellular growth, survival, and cell cycle, the dysregulation of which is frequently detected in human cancers (35). For example, microRNA-27a (miR-27a) downregulates FBXW7 expression by binding to its 3’-untranslated region (3’-UTR), and promotes OSCC cell proliferation *via* the PI3K/AKT axis (36). The serine/threonine kinase mTOR is capable of interacting with both upstream and downstream proteins of the PI3K/AKT pathway, which performs an indispensable role in orchestrating metabolism, cellular proliferation, and survival (37). FBXW7 deficiency results in elevated mTOR and phosphorylated mTOR (p-mTOR) protein levels, supporting that the turnover of mTOR is controlled by FBXW7 *via* targeted ubiquitination and degradation (38). Ectopic expression of the oncogene family with sequence similarity 83, member D (FAM83D) facilitates breast cancer cell proliferation partly through the accumulation of mTOR by reducing the expression level of FBXW7 (39).

As a substrate of FBXW7-dependent ubiquitination and proteolysis, the Notch signaling pathway has significant influences on cellular differentiation, proliferation, and apoptosis (40), while the dysregulated Notch signaling is also linked to oncogenesis of both solid tumors and leukemias (41, 42). Liu et al. have revealed that the serine/threonine/tyrosine interacting protein (STYX) accelerates endometrial cancer cell proliferation and suppresses apoptosis partly through the Notch/mTOR pathway by downregulating the expression of FBXW7 (43). The relationship between Notch and mTOR has been explored, presumably that the HES1 protein activated by Notch suppresses PTEN transcription, subsequently promoting the phosphorylation of AKT and mTOR activation (44).

In gastric cancer (GC), the downregulated level of FBXW7 attenuated its impact on growth arrest and apoptosis (45). One possible mechanism has elucidated that the transcription factor growth factor independence 1 (GFI1) promotes cell proliferation partly by means of suppressing transcription of Gastrokine-2 (GKN2) (46). Overexpression of GKN2 in GC cell lines suppresses GC cell proliferation through the downregulation of the JAK/STAT signaling pathway. Noteworthily, GFI1 mutant on S94A/S98A inhibits its phosphorylation mediated by GSK3β and ubiquitination by FBXW7, eventually facilitating tumor progression.

### 2.2 Resisting Cell Death

#### 2.2.1 Apoptosis

The apoptotic machinery comprises both upstream regulators and downstream effectors, the latter eliciting a series of cascades pointed to the death program. The signals between regulators and effectors are transmitted by the so-called “apoptotic trigger” in the control of the B cell lymphoma 2 (BCL2) protein family including both pro- and anti-apoptotic regulatory proteins (1, 2).

By virtue of flow cytometry, apoptosis assays, and caspase 3/7 activity assays, researchers found that overexpression of FBXW7 accelerates GC tissue apoptosis through inducing the ubiquitination and degradation of RhoA (45), which is known to promote survival by triggering the expression of BCL2 gene (47). MCL-1, a member of BCL2 protein family, functions as a crucial anti-apoptotic regulator in cellular differentiation and apoptosis (48), which is overexpressed in multiple malignancies giving rise to chemotherapeutic resistance (49–51). It has been reported that FBXW7 regulates apoptosis cascades through ubiquitination and degradation of MCL-1. In T-ALL cell lines, FBXW7 deficiency confers resistance to chemotherapy, which can be reversed by FBXW7 restoration or MCL-1 knockout, implicating that MCL-1 promotes FBXW7-deficient cells to escape from apoptosis (52, 53). Likewise, FBXW7 mutations also mediates chemoresistance to some solid tumors such as CRC (24, 54), squamous cell carcinoma (55), and BC (56), which attributes to the downregulation of MCL-1. Meanwhile, favorable response to chemoradiotherapy is correlated with a high level of FBXW7, which is in parallel with a low level of MCL-1 (57).

In terms of targeting FBXW7 itself, the downregulation of prolyl isomerase Pin1 contributes to upregulation of FBXW7 and ensuing destruction of MCL-1, which potentiates the toxicity of sorafenib to prevent cell proliferation and induce apoptosis in hepatocellular carcinoma (HCC) (58). Similarly, the alkaloid lycorine hydrochloride (LH) has been reported to upregulate the level of FBXW7 as well as destabilize MCL-1, facilitating BCL2-drug-resistant GC cell apoptosis and inhibiting proliferation (59). Moreover, tyrosine kinase inhibitors (TKIs) give rise to GSK3β-dependent phosphorylation of MCL-1, translocation of MCL-1 to nucleus, as well as FBXW7-mediated degradation through targeting PI3K/AKT signaling, which overcome the resistance to targeted therapy in non-small cell lung cancer (NSCLC) (60). In addition, directly targeting MCL-1 with small molecular MCL-1 inhibitors exhibits potent efficacy such as S63845, AZD5991, and AMG176, sensitizing CRC cells to treatment of the targeted drug regorafenib by virtue of restoring apoptotic response (61). Collectively, accumulated evidence has deciphered MCL-1’s role in therapeutic resistance, in combination with novel strategies to dampen therapeutic resistance by targeting MCL-1, FBXW7, and their upstream proteins.

The mitochondria integrate an array of proapoptotic signals, further releasing proapoptotic signaling proteins such as cytochrome c to trigger the progression of a cascade of apoptotic caspases. FBXW7 facilitates apoptosis of glioblastoma cells through the ubiquitination and proteolysis of histone deacetylase 7 (HDAC7) (62), which localizes to the inner membrane of mitochondria and will be released into and sequestered within the cytoplasm in response to the initiation of apoptotic cascades (63).

c-MYC has been reported to have fundamental impacts on cell cycle progression as well as programmed cell death (64–66). The Aurora B kinase (AUKRB) impedes the phosphorylation of GSK3-3β and subsequent FBXW7-mediated degradation, rendering the accumulation of c-MYC and further promoting the progression of T-ALL (67). Nevertheless, the AUKRB inhibitor can reverse leukemogenesis and induce apoptosis due to the destabilization of c-MYC. Furthermore, c-MYC influences apoptosis partly through the p53 signaling pathway (68). The FBXW7 deficiency in the T-cell lineage of mice results in the accumulation of c-MYC, which promotes p53 expression, and further cell cycle arrest and apoptosis. The abnormalities can be reversed by the suppression of p53 (69). The development of glioblastoma can be ascribed to p53 mutations, which promotes the accumulation of c-MYC through the depression of FBXW7 and prevents apoptosis (70).

In pancreatic cancer tissues, FBXW7 induces apoptosis by inhibiting the binding of nuclear receptor subfamily 4 group A member 1 (NR4A1) to stearoyl-CoA desaturase 1 (SCD1) promoter, thus suppressing the transcription of SCD1 (71). SCD1 inhibition has been reported before to inactivate the AKT signaling, seen in higher rates of cell apoptosis in human lung adenocarcinoma (72). FBXW7 promotes apoptosis of BC cells through the ubiquitination and proteolysis of the oncoprotein metadherin (MTDH) (73), whose expression is related to various pathways, for example, the AKT signaling pathway (74). Furthermore, the inhibition of MTDH gives rise to apoptosis of cancer cells probably due to the downregulation of the AKT signaling (75, 76).

#### 2.2.2 Autophagy

Autophagy is recognized as an evolutionarily conserved, intracellular degradation process, capable of delivering cytoplasmic materials to lysosomes (77) and regulating energy homeostasis as well as cellular renovation (78). Based on the differences of physiological functions and delivering modes, autophagy can be subdivided into mainly three types: macroautophagy that is thought to be the major type, microautophagy, and chaperone-mediated autophagy (CMA) (77). Dysregulated autophagy is involved in the development of multiple cancers, exerting a bifunctional role in suppressing benign tumor development and inducing cancer progression (79). In addition, autophagy can be induced during treatment of drug-sensitive cancers, resulting in drug resistance and cancer relapse (80). In general, inhibition of autophagy has been proposed as an effective therapeutic intervention (81).

The central metabolism modulator mTOR could be directly regulated by FBXW7 through ubiquitination and subsequent degradation (38, 82). mTOR is an essential inhibitory regulator of autophagy in response to growth factors as well as abundant nutrients (77), especially mTOR complex 1 (mTORC1), which regulates autophagy both in the process of initiation and transcription (83). Given the pivotal suppressive functions of mTOR in modulating autophagy, we propose the possible metabolic crosstalk between FBXW7 and autophagy. Perifosine is an oral alkylphospholipid with antitumor activity, whereas the pharmacological efficacy can be partly counteracted by inhibiting the mTOR axis dependent on GSK3/FBXW7 and inducing prosurvival autophagy (84). SHOC2, a RAS/ERK activator and a substrate of FBXW7, selectively combines with Raptor to impede Raptor-mTOR binding and subsequent mTORC1 activity, contributing to the stimulation of autophagy and acceleration of cancer proliferation (27, 85). Furthermore, there is a negative feedback loop between mTORC1 and RAS/ERK pathways, both under the regulation of FBXW7 targeting SHOC2 (85).

In addition to tumorigenesis, autophagy is also integral to therapeutic effects, capable of mediating resistance or sensitivity in response to chemotherapy agents (86). Wang et al. demonstrated that the microRNA-223 (miR-223) overexpression downregulates the level of FBXW7 protein, resulting in the activation of autophagy and cisplatin resistance of non-small lung cancer cells (NSLCCs) (87). Furthermore, this result indicates that targeting microRNAs can be of great value to inhibit autophagy and attenuate chemoresistance, which will be discussed in detail in later sections.

### 2.3 Inducing Angiogenesis

Growing malignant tumors are consistently endowed with the identity of neovascularization, referred to the vigorous and persistent growth of new capillaries (88). Angiogenesis plays a pivotal part in supplying essential oxygen and nutrients for solid tumor to satisfy ever-growing metabolic demands. Hypoxia-inducible factor (HIF) is a heterodimeric transcription factor, with the ability to trigger a large scale of pro-angiogenic factor expressions containing vascular endothelial growth factor (VEGF), VEGF-R1, and VEGF-R2, plasminogen activator inhibitor‐1 (PAI-1), matrix metalloproteinase-2 (MMP-2) and MMP-9, Angioprotein-1 (Ang-1) and Ang-2, as well as Tie-2 receptor (89, 90). In particular, the HIF-1α pathway is acknowledged to mainly regulate vasculature formation (89). GSK-3β/FBXW7-mediated ubiquitination and degradation is shown to influence angiogenesis and cell metastasis by decreasing the level of HIF-1α (91), in contrast to ubiquitin-specific protease 28 (USP28) which abrogates FBXW7-dependent degradation (92). miR-182, as previously mentioned, figures as an oncogenic miRNA by targeting its downstream gene FBXW7 in BC, capable of accelerating HIF-1α/VEGF-A-induced angiogenesis under hypoxia condition (93). miR-144, enriched in tumor-derived extracellular vesicles (EVs) from nasopharyngeal carcinoma (NPC), is demonstrated *in vitro* and *in vivo* to promote angiogenesis by downregulating FBXW7 and promoting HIF-1α-induced VEGF-A expression (94). Notably, FBXW7 can inactivate β-catenin pathway to influence the expression of VEGF-A, ultimately suppressing angiogenesis of ovarian cancer cells (95).

The Notch signaling pathway is crucial to vascular construction reflected in regulation of sprouting tip cells, endothelial cell development, as well as arterial differentiation (96). In melanoma, loss of FBXW7 is relevant to enrichment of Notch1, enhanced expression of Notch1 target genes, as well as acceleration of angiogenesis, which vividly elucidates FBXW7 as a critical suppressor of angiogenesis partly through degrading its substrate Notch1 and influencing its downstream signaling (97). In view of the pivotal role of FBXW7 in regulating angiogenesis, it is ponderable to investigate the underlying efficacy of dampening angiogenesis by targeting FBXW7 and its downstream factors.

### 2.4 Activating Invasion and Metastasis

Epithelial–mesenchymal transition (EMT) refers to epithelial cells’ acquisition of mesenchymal phenotypes, and is a highly conserved cellular program that is crucial to embryonic development and malignant progression such as migration, invasion, stemness, and therapeutic resistance (98–101). FBXW7 expression abundance is relevant to tumor clinicopathologic features, and FBXW7 is capable of dampening EMT process partly through downregulating EMT upstream transcription factors such as Snail 1 (45, 102) and zinc-finger E-box-binding homeobox 1 (ZEB1) (45), whereas the downregulation of FBXW7 expression reverses its inhibitory role. Research reveals that FBXW7 is involved in renal cell carcinoma (RCC) cell invasion and metastasis by virtue of suppressing EMT, which has great potential for future therapeutic targets (103, 104). Moreover, non-coding RNAs regulate tumor properties of invasion and metastasis *via* EMT in part through interacting with FBXW7. MiR-27a overexpression in human BC induces cell migration and EMT dependent on its target gene FBXW7, while ectopic expression of FBXW7 attenuates the properties of invasion and metastasis partly associated with EMT (105). Cancer susceptibility candidate 2 (CASC2), a long non-coding RNA (lncRNA) downregulated in HCC cell lines, can inhibit tumorigenesis by means of functioning as a competing endogenous RNA (ceRNA) for miR-367 and weakening its pro-metastatic effects through targeting FBXW7 (106).

Consequently, this pathway provides insights into potential targeted therapies. It is well-known that FBXW7 controls various cellular processes by regulating its substrates, of which c-MYC and Notch participate in the promotion of EMT process (107, 108). The ATPase Thyroid hormone receptor interactor 13 (TRIP13), usually overexpressed in a myriad of human cancers, promotes glioblastoma migration and invasion by decreasing the transcription of FBXW7 and attenuating its inhibitory effects on c-MYC (109). Dysregulated FBXW7 appears to be a prognostic hallmark of HCC, exhibiting that lower expression corresponds to worse progression and survival. Moreover, FBXW7 is capable of modulating HCC invasion and metastasis *via* FBXW7/Notch1 axis (23).

In addition to EMT process, there are multiple underlying mechanisms accounting for the invasion, migration and metastasis of cancer cells. For example, mitogen-activated protein kinase/extracellular signal–regulated kinase (MAPK/ERK) pathway appears to be involved in melanoma tumorigenesis, for the treatment of MAPK/ERK kinase (MEK) inhibitors significantly reverses FBXW7 knockdown-induced cell migration (110). Enhancer of zeste homolog 2 (EZH2), a substrate of FBXW7, figures as an oncogenic protein facilitating invasion and metastasis of pancreatic cancer cells, which can be abrogated by CDK5/FBXW7-dependent degradation (111). Cai et al. reported that FBXW7 participates in regulating RCC metastasis partly through modulating expression of MMP-2, MMP-9, and MMP-13 (103), which have been disclosed to degrade extracellular matrix (ECM) protein to accelerate cancer metastasis (112).

### 2.5 Reprogramming Energy Metabolism

**Figure 3:**
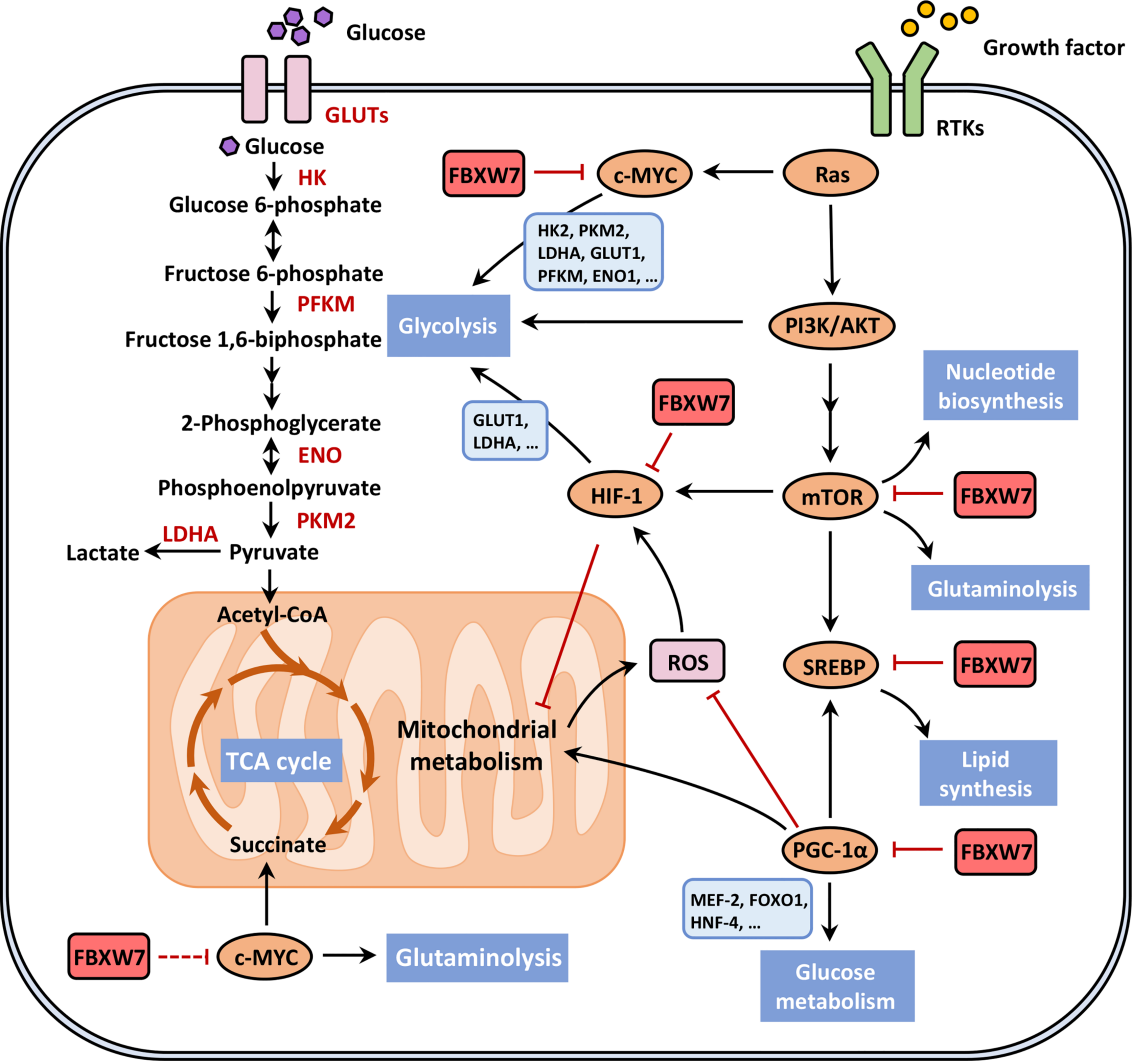
FBXW7 participates in the reprogramming energy metabolism. The rewired metabolic network favors cancer cells to meet essential energy, anabolic and redox demands on acquired nutrients during early stages of cancer development, where FBXW7 modulates metabolic reprogramming through ubiquitin-dependent degradation of crucial metabolic molecules, such as mTORC1, SREBP, HIF-1, c-MYC and PGC-1α, their relationship seen in red solid blocking arrows. We are awaiting the potential roles of FBXW7 in regulating tricarboxylic acid (TCA) cycle and glutamine metabolism through interacting with c-MYC, so the red dotted line blocking arrow is shown in the lower left part of the figure. This figure was adapted from DeBerardinis and Chandel (118). ENO, enolase; FOXO1, fork head box O1; GLUT, glucose transporter; HK, hexokinase; HNF-4, hepatic nuclear factor-4; LDHA, lactate dehydrogenase A; MEF-2, myocyte enhancer factor-2; PFKM, phosphofructokinase; PKM2, pyruvate kinase M2; ROS, reactive oxygen species; RTK, receptor tyrosine kinase.

Under the condition of hypoxia or even adequate oxygen supply, cancer cells choose inefficient aerobic glycolysis rather than oxidative phosphorylation (113), giving rise to increased glucose consumption, high-speed ATP production, as well as transformation of glycolytic pyruvate to lactate, widely known as “the Warburg effect” (114). The rewired metabolic network produces intermediates, for example, figuring in the process of glycolysis or tricarboxylic acid (TCA) cycle, not only favoring cancer cells to meet essential energy, and anabolic and redox demands on acquired nutrients during early stages of cancer development (115), but also supporting cancer malignant progression especially metastasis and therapeutic resistance in later stages (116, 117) based on the resistance to oxidative stress. The circuit of energy reprogramming involves a series of signaling pathways (118), where FBXW7 participates in the regulation of crucial metabolic molecules (**Figure 3**).

Growth factors trigger the PI3K/AKT axis as well as the downstream mTOR pathway, inducing the glycolytic flux and fatty acids (FAs) production due to activation of HIF and sterol regulatory element binding protein (SREBP), respectively. The mTOR pathway performs an indispensable role in nutrient metabolism, energetic regulation, and promotion of cancer cell survival (119). mTORC1 enhances the transcription of glycolysis-related genes (120), and mTOR was proven to be a crucial activator of Warburg effect (121). Evidence has shown that the activation of mTORC1 promotes nucleotide synthesis as well as glutaminolysis (118). Various studies report that FBXW7 targets and degrades mTOR through ubiquitin–proteasome pathway (38, 122, 123). Fructose-1,6-bisphosphatase (FBP1) was reported to promote the autoubiquitination of FBXW7, then resulting in lower levels of mTOR and subsequently deregulated glycolysis in NPC cells (122).

In addition to the functions of HIF-1 in angiogenesis, HIF-1 exhibits a pivotal role in the aerobic glycolysis under hypoxic conditions, including the induction of glucose transporter 1 (GLUT1) and critical glycolytic enzymes such as lactate dehydrogenase A (LDH-A) (124). Furthermore, HIF-1 suppresses the functions of mitochondria to regulate the Warburg effect, comprising the inactivation of TCA cycle, enzymes, and microRNAs associated with the mitochondrial process, as well as the decrease of activated mitochondria. Under the circumstance of hypoxia, HIF-1α is negatively regulated by FBXW7-mediated ubiquitination and proteolysis in human ovarian cancer cells, proposing an underlying direction for energy reprogramming and angiogenesis (91). The oncoprotein TAR (HIV-1) RNA binding protein 2 (TARBP2) can elevate the stability of HIF-1α by decreasing its ubiquitination level through decreasing the E3 ligases activity including FBXW7 in BC cells (125).

SREBPs, a family of membrane-bound transcription factors (126), are able to induce the expression of genes associated with lipid metabolism (127–129) and multiple cellular processes (130), which can be subdivided into three major members encoded by two distinct genes (126). SREBPs translocate to the nucleus in an active form and regulate corresponding gene expressions, and are degraded rapidly by the ubiquitin–proteasome pathway (131). In recent years, researchers have deciphered the role of FBXW7 in lipogenesis related with SREBPs, whereas the precise regulatory mechanisms remain to be elucidated. Dependent on the phosphorylation of Cdc4 phosphodegron (CPD) motifs, FBXW7 interacts with and degrades SREBP1 and SREBP2 in control of lipid metabolism (132, 133), reflected in the elevated cholesterol and FAs synthesis as well as the enhanced uptake of LDL in FBXW7-/- cells (132). miR-182, originated from a single primary transcript (134), is emerging as an oncogenic role in various malignancies (135, 136). miR-182 was reported to target FBXW7 and indirectly enhance the accumulation of nuclear SREBPs; subsequently, lipid synthesis was accelerated by stimulation of SREBP-targeted genes (137). To meet the demand for rapidly excessive proliferation, cancer cells rely on glucose and glutamine for *de novo* lipogenesis responsive to dysregulated SREBP pathways (138). In an experiment, different cancer cells were treated with inhibitors of mTOR complex 2 (mTORC2) and then detected to express decreased mature SREBP1 (mSREBP1). It was unraveled that mTOR2 suppresses the GSK3/FBXW7-mediated ubiquitination of SREBP1 and serves as a positive regulator of SREBP1-related genes and lipogenesis (139). Likewise, protein arginine methyltransferase 5 (PRMT5) facilitates *de novo* lipogenesis by virtue of epigenetic modification of SREBP1a and blockade of GSK3/FBXW7-mediated ubiquitin–proteasome pathway (140).

c-MYC exerts its roles in various biological processes such as proliferation, metabolism, differentiation, and transformation as mentioned before (28). c-MYC modulates almost all glucose metabolism genes, including hexokinase 2 (HK2), pyruvate kinase M2 (PKM2), LDHA, GLUT1, phosphofructokinase (PFKM), and enolase 1 (ENO1) (141, 142), promoting the development of glycolysis. Additionally, c-MYC plays a significant part in FA biosynthesis, glutaminolysis, serine metabolism, and mitochondrial metabolism. Several studies have demonstrated that FBXW7 interacts with and ubiquitylates c-MYC, and phosphorylation of c-MYC on Thr-58 and Ser-62 is essential for proteasome-dependent degradation (31). FBXW7 dramatically suppresses glucose metabolism and reduces the 18F-FDG uptake of pancreatic cancer cells through regulating the expression of thioredoxin binding protein (TXNIP) in a c-MYC-dependent manner, whose regulatory mode can be summarized into the FBXW7/c-MYC/TXNIP axis (114). PRMT5 epigenetically regulated the expression of FBXW7, contributing to elevated c-MYC levels and subsequently enhanced glycolysis and proliferation of pancreatic cancer cells (22). Reversely, dioscin treatment enhanced the interaction between FBXW7 and c-MYC, which leads to inhibition of HK2, a crucial enzyme catalyzing glucose phosphorylation and hence the suppression of glycolysis (143). Likewise, Tanshinone IIA (Tan IIA) suppresses HK-mediated glycolysis of oral squamous cell carcinoma (OSCC) cells by promoting FBXW7-mediated degradation of c-MYC and inhibiting the AKT-c-MYC signaling, exhibiting its natural antitumor activity (144).

In addition, FBXW7 also ubiquitinates and degrades PPARγ coactivator-1α (PGC-1α), which serves as a critical energy metabolism regulator, coordinating mitochondrial biogenesis and oxidative phosphorylation (145). Outside the mitochondrion, PGC-1α also modulates lipid and glucose metabolism through the interaction with nuclear factors, such as SREBP1, myocyte enhancer factor-2 (MEF-2), fork head box O1 (FOXO1), and hepatic nuclear factor-4 (HNF-4) (146). In human melanoma cell lines, the microphthalmia-associated transcription factor (MITF) directly controls PGC-1α expression; hence, the MITF/PGC-1α axis can regulate mitochondrial oxidative phosphorylation and rescue the oxidative stress of reactive oxygen species (ROS) (147, 148). FBXW7 expression was detected downregulated, which is correlated with elevated expression of mitochondrial functional genes as well as poor prognosis of melanoma patients, dependent on its downstream MITF/PGC-1α pathway satisfying the metabolic needs of tumor cells (149). In contrast to the aforementioned tumorigenic functions of PGC-1α, the diminution of PGC-1α indirectly by HIF renders metabolic reprogramming, accelerating tumorigenesis and the resistance to chemotherapies in RCC cells (150). The dual roles of PGC-1α in metabolic reprogramming remain elusive and require to be explored. In addition, FBXW7 mutations in CRC cells enhance the mitochondrial gene expression, in which the so-called “metabolic reprogramming” points to oxidative phosphorylation and is possessed with metabolic vulnerabilities (151).

### 2.6 Avoiding Immune Destruction

The consistently alerted immune system has been monitoring and eliminating cells and tissues from forming and progressing early-stage neoplasms, terminal tumors, and micrometastases. According to this theory of immune surveillance, solid tumors avoid immune eradication through evading immune detection or curtailing the damage of immunological killing (2), both of which can be realized through presenting tumor antigens resistant to immune effectors (also called “immunoediting”) and the progressive formation of immunosuppressive microenvironment (152).

Tumor-associated macrophages (TAM), the macrophages that are characterized as immunosuppressive infiltrating tumors, promote tumor progression through subversion of genetic stability, nurturing cancer stem cells (CSCs), promoting metastasis, and restraining adaptive immune response (153). Driven by cytokines derived from tumors and T cells, macrophages polarize into classically activated macrophages (also called M1) and alternatively activated macrophages (also called M2), in which TAM mainly belong to the latter, showing anti-inflammatory and pro-tumorigenic functions (154). FBXW7 is capable of restricting cancer progression by haltering immunosuppressive niche and immune evasion. Myeloid cell-specific ablation of FBXW7 enhances tumor proliferation by decreasing its ubiquitination on c-MYC, which promotes TAM polarization (155).

Moreover, in a triple-negative BC (TNBC) model, low-expressed E74-like transcription factor (ELF5) downregulates the level of FBXW7, attenuating its degradation on interferon-γ receptor 1 (IFNGR1). Afterwards, the triggered intrinsic interferon-γ (IFN-γ) pathway facilitates immunosuppressive neutrophil aggregation, accompanied by programmed death ligand 1 (PD-L1) upregulation, which not only promotes tumor proliferation and metastasis but also appears to accelerate CD8+ T cell exhaustion favoring immune evasion. Therapeutic drugs targeting IFNGR1 and PD-L1 potentiate antitumor efficacy in TNBC patients, providing new insights into underlying targets for immunotherapies (156). FBXW7 inhibits the transcription of Toll-like receptor 4 (TLR4) signaling by degrading CCAAT/enhancer binding protein delta (C/EBPδ) phosphorylated by GSK3β, detected as transcriptional activator of Tlr4 genes (157). Reversely, C/EBPδ also represses the expression of FBXW7 (158), thus forming a negative feedback cycle through which FBXW7 inhibits inflammatory responses and TLR4-associated immune evasion (159). In addition, high expression of miR-101 and miR-26a are detected in ovarian cells, which downregulate EZH2 and confer functional inactivation of effector T cells in the absence of glucose. In mechanism, the histone methyltransferase EZH2 triggers notch signaling by repressing its suppressor Numb and FBXW7, maintaining effector T-cell survival and poly-functions (160).

### 2.7 Genome Instability and Mutation

Genomic alterations promote the acquisition of other hallmarks of cancer cells, prioritizing subclone of cells with superior mutant genotype to expand progressively and play a leading role within the local environment. The maintenance of genome stability requires delicate modulation of DNA replication in the cell cycle process, precise detection, and repairing any defects of DNA.

As a substrate of FBXW7, cyclin E, a nuclear protein that interacts with and activates cyclin-dependent kinase 2 (CDK2) to phosphorylate proteins, facilitates G_1_/S phase transition and is degraded at the boundary of the S/G_2_ phase, whereas the overexpression of cyclin E has been detected in human cancer cells associated with cell cycle deregulation and chromosome instability (CIN) (161, 162). FBXW7 mutations lead to the aberrant accumulation of its substrate cyclin E in CRC samples, conferring abnormality of chromosome congression during metaphase as well as ensuing chromosome transmission, implicating the role of FBXW7/cyclin E axis in regulating CIN (163). An *in vivo* study generated a mouse model to disturb the regulation of cyclin E relying on FBXW7, which was found fragile in response to hematologic stress and induced CIN, ultimately pointing to fatal T-cell malignancies (164). Additionally, cyclin E/CDK2 overactivation due to FBXW7 deficiency could induce hyper-phosphorylation of the centromere protein CENtromere Protein A (CENP-A) at the N-terminal Ser18 site, thus promoting chromosome missegregation and micronucleus formation, demonstrating that the FBXW7-dependent cyclin E is related with CIN *via* centromere dysfunction (165, 166).

Several essential proteins and microRNAs existing in or interacting with the FBXW7/cyclin E pathway may be underlying targets to overturn genomic instability and disrupt tumorigenesis. Both functioning as the upstream proteins of FBXW7, the integrator protein TRIP-Br coordinates with the transcription regulator early 2 factor (E2F) to regulate S phase execution and maintain genomic stability partly *via* the FBXW7/cyclin E axis (167). MiR-223 targets the 3’-untranslated region of FBXW7 to regulate cyclin E activity and cyclin E-mediated genomic stability, whereas miR-223 expression can be responsive to the acute changes of cyclin E expression, thus forming a negative feedback circuit among miR-223, FBXW7, and cyclin E (168). Meanwhile, Aurora-A amplification was detected frequently in CRC tissues and significantly correlated with a fractional allelic loss (FAL) score, so targeting Aurora-A kinase in the FBXW7/cyclin E pathway can be a potential CRC therapy associated with CIN (169). The excessive activation of the oncogenic ERK1/2 MAP kinase (MAPK) pathway results in the downregulation of FBXW7β both in epithelial cell lines and in the intestine tissue of a transgenic mouse model, conferring the stabilization of Aurora-A, abnormal cell division, and polyploidization. Consequently, the ERK1/2/FBXW7/Aurora-A axis is associated with aneuploidy and epithelial malignancies, and the MAPK can be a good candidate for target therapy in the not-too-distant future (170). Under the governance of p53-dependent transcription regulation, FBXW7 protects epithelial cells from DNA damage and malignant progression presumably through its substrate such as cyclin E, Aurora A, or c-MYC, and p53 heterozygosity combined with FBXW7 loss can confer aneuploidy as well as epithelial cancers (171). Another *in vitro* study generated adenocarcinomas exhibiting aneuploidy, which were derived from FBXW7-/-; p53-/- cell lines, implicating that ablation of FBXW7 and p53 synergistically promotes the occurrence of CIN and CRC tumorigenesis (172).

As major components of surveillance mechanism, cell cycle checkpoints ensure the order, integrity, and fidelity of crucial events throughout the cell cycle, monitoring cell size, the responses to DNA replication, and DNA damage (173). Under the stress of UV irradiation, FBXW7α induces proliferation arrest in the S phase through interacting with and ubiquitinating polo-like kinase 1 (PLK1) that functions in eukaryotic cytokinesis, showing the role of the FBXW7/PLK1 axis in the regulation of the intra-S-phase DNA-damage checkpoint (174). The mice bearing P48-Cre; LSL-KRAS^G12D^; FBXW7^fl/fl^ (KFC^fl/fl^) mutations exhibited progressive pancreatic tumorigenesis with elevated numbers of micronuclei, as a result of Yes-associated protein (YAP) overexpression due to FBXW7 deficiency (175). The role of oncogenic protein YAP in driving genomic instability has also been established in a medulloblastoma mouse model. Mechanically, YAP triggers the expression of insulin-like growth factor 2 (IGF2) and ensuing activation of the PI3K/AKT pathway after radiation, thus avoiding DNA repair and inactivating the checkpoint regulators of ATM and CHK2, the latter inhibiting the G_1_/S and G_2_/M checkpoints (176).

In terms of repairing machinery, FBXW7 participates in the Fanconi anemia (FA) pathway that deals with DNA interstrand cross-link (ICL) repair through ubiquitinating and degrading the crucial component of the FA pathway, FAAP20 (177). Additionally, FBXW7 promotes nonhomologous end-joining (NHEJ) that resolves DNA double-strand breaks (DSBs) in mammalian cells *via* FBXW7α-dependent XRCC4 polyubiquitylation (178).

## 3 Perspectives: Potential Therapeutic Strategies

**Figure 4:**
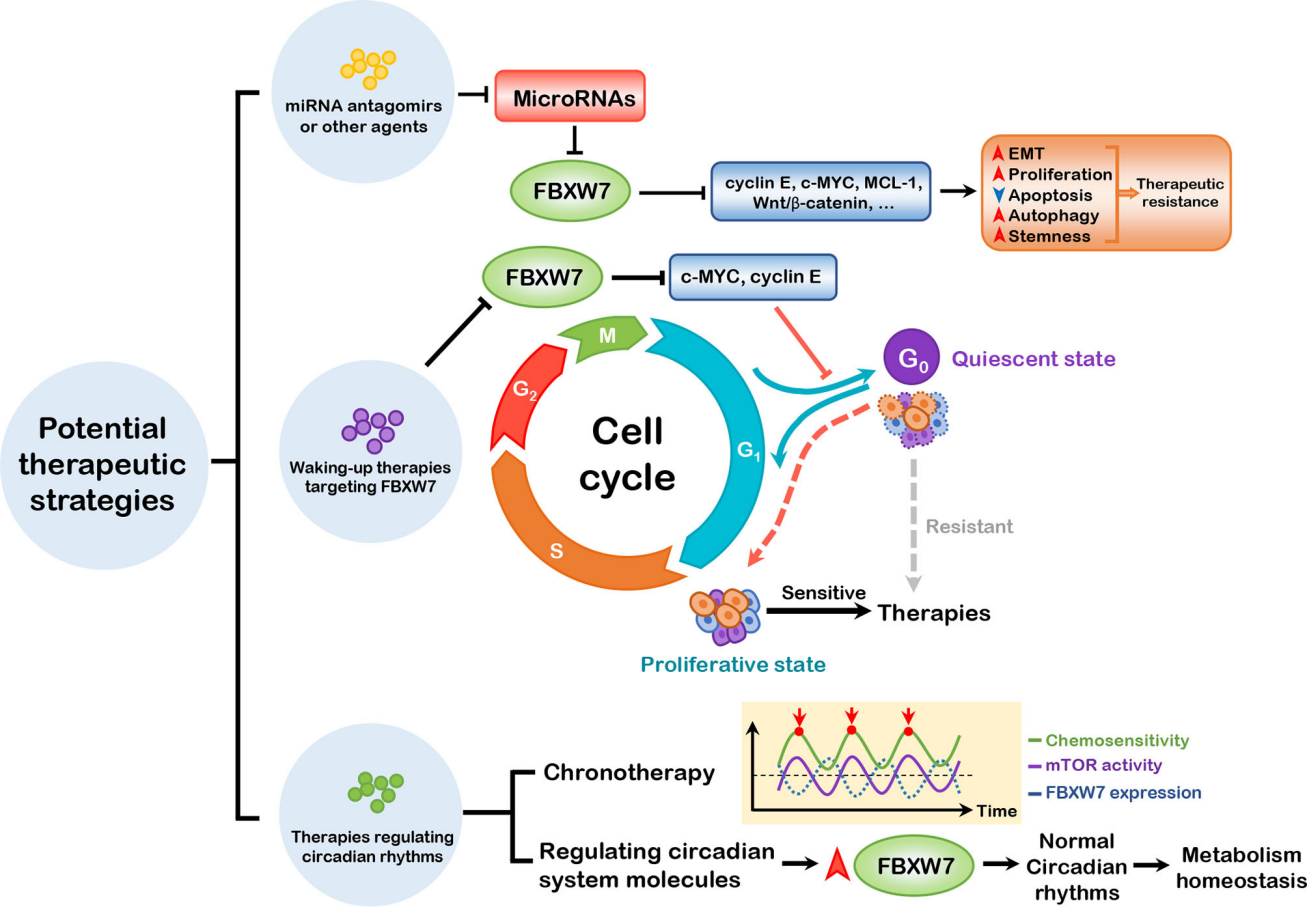
Three potential therapeutic strategies. miRNA antagomirs or other agents can be administered to block the suppression of FBXW7 expression by miRNAs, thus inhibiting the substrates of FBXW7, which promote EMT, proliferation, autophagy, and stemness and dampen apoptosis to induce therapeutic resistance. Waking-up therapies can be applied to prevent exit of cells from cell cycle regulated by FBXW7, thus waking up cancer stem cells from quiescent to proliferative state. Based on the role of FBXW7 in regulating circadian rhythms, it is ponderable to introduce chronotherapy and elevating FBXW7 expression to attain better therapeutic efficacy. EMT, epithelial–mesenchymal transition; MCL-1, myeloid cell leukemia-1.

Therapeutic resistance remains one of the thorniest obstacles in the course of cancer treatment, which are associated with relapse and poor prognosis of patients. Tumors can be resistant due to a single or a combination of biological determining factors, involving intrinsic factors such as tumor burden and growth kinetics, tumor heterogeneity, physical barriers, tumor microenvironment, and undruggable genomic drivers (e.g., c-MYC and TP53), and extrinsic factors such as selective therapeutic pressure. The discussed hallmarks of cancer associated with FBXW7 present necessary insights to better understand the relationship between FBXW7 and therapeutic resistance, emphasizing the urgency of establishing novel targets for precise therapy. We propose three potential strategies against therapeutic resistance as follows (seen in **Figure 4**).

### 3.1 Targeting MicroRNAs to Enhance Therapeutic Sensitivity of Cancers

miRNAs refer to short endogenous non-coding RNAs with a length of ∼22 nucleotides, targeting at the complementary site of mRNAs to mediate the degradation or repression of different genes that modulate development, apoptosis, differentiation, and even tumorigenesis (179). Frequently altered miRNAs in diseases make it possible to target these molecules in the form of miRNA mimics or anti-miRs for cancer therapy, combined with the effective application of RNA-delivering constructs (180). An increasing number of *in vitro* and *in vivo* studies has implicated that microRNAs can regulate cancer progression and therapeutic resistance by virtue of interacting with FBXW7, such as miR-223, miR-363, miR-27b-3p, and miR-92a-3p (181–184).

In addition to functioning in the hematopoietic system, miR-223 is in close relationship with multiple malignancies, given that miR-223 targets (e.g., IGF-1 receptor and stathmin 1) have been disclosed to impact the hallmarks of cancer, involving proliferation, invasion, metastasis, etc. (185). *In vitro* and *in vivo* studies have substantiated that the upregulated expression of miR-223 dampens therapeutic sensitivity in several cancer types, such as T-ALL, GC, NSCLC, HCC, and CRC (seen in **Table 1**). For example, miR-223 was detected upregulated in cisplatin-resistant GC cell lines, and the elevated expression of miR-223 inhibits cisplatin sensitivity of GC cells *in vitro*; these results can be explained by miR-223 target protein FBXW7, which regulates cell cycle and apoptosis presumably through ubiquitin-mediated proteolysis of MCL-1 (187).

**Table 1:** MicroRNAs regulate therapeutic resistance *via* targeting FBXW7 and the possible mechanisms.

miRNAs	Cancer types	*In vitro*/*in vivo*	Expression pattern	Therapies induced resistance by miRNAs	Possible mechanisms *via* targeting FBXW7	Potential strategies targeting miRNAs for elevating therapeutic sensitivity	References
miR-223	T-ALL	*In vitro* and *in vivo*	Upregulated	GSI IX(DAPT)	GSI induces increased C/EBPα expression, which activates miR-223 expression, thus leading to FBXW7 loss, whereas the exact mechanism of FBXW7’s impacts on therapeutic resistance is not mentioned	Inhibitors of NF-κB signaling, notch1 and notch3; miR-223 antagomir	(181)
miR-223	GC	*In vitro*	Upregulated	Trastuzumab	Upregulated miR-223 induces trastuzumab resistance by suppressing apoptosis though the FBXW7/MCL-1 axis	miR-223 antagomir	(186)
miR-223	GC	*In vitro*	Upregulated	Cisplatin	miR-223 alteration promotes cisplatin resistance by regulating the G1/S cell cycle and apoptosis though interacting with FBXW7	miR-223 antagomir	(187)
miR-223	GC	*In vitro*	Upregulated	Doxorubicin	miR-223 derived from TAM targets FBXW7 to induce EMT process	miR-223 antagomir	(188)
miR-223	NSCLC	*In vitro* and *in vivo*	Upregulated	Cisplatin	miR-223 targets FBXW7 to enhance cisplatin-induced autophagy	miR-223 antagomir	(87)
miR-223	NSCLC	*In vitro*	Upregulated	Erlotinib	miR-223 promotes proliferation and inhibits apoptosis by inhibiting FBXW7	Inhibitors of the Akt and Notch pathways; MiR-223 antagomir	(189)
miR-223	HCC	*In vitro*	Upregulated	Sorafenib	The exact mechanism of FBXW7’s impacts on therapeutic resistance is not mentioned	miR-223 antagomir	(190)
miR-223	CRC	*In vitro*	Upregulated	Doxorubicin	miR-223 induces EMT process though interacting with FBXW7	miR-223 antagomir	(191)
miR-363	GC	*In vitro*	Upregulated	Docetaxel + Cisplatin + 5-FU (DCF) regimen	miR-223 promotes cell proliferation and DCF resistance by targeting FBXW7	miR-363 antagomir	(182)
miR-27b-3p	MM	*In vitro*	Upregulated	Not mentioned	miR-27b-3p induced by MM-derived exosomes stabilize MCL-1 to suppress apoptosis by targeting FBXW7	miR-27b-3p antagomir	(183)
miR-92a-3p	CRC	*In vitro* and *in vivo*	Upregulated	5-FU/L-OHP	Exosomal miR-92a-3p from CAFs promote cell stemness and EMT, as well as inhibit cell apoptosis by targeting FBXW7	miR-92a-3p antagomir	(184)
miR-500a-3p	GC	*In vitro* and *in vivo*	Upregulated	Cisplatin	miR-500a-3p promotes cell stemness by targeting FBXW7	miR-500a-3p antagomir	(192)
miR-19b	CRC	*In vitro* and *in vivo*	Upregulated	Radiation therapy	miR-19b regulates the FBXW7/Wnt/β-catenin axis to promote stemness properties	miR-19b antagomir	(193)
miR-25-3p	Glioblastoma	*In vitro* and *in vivo*	Upregulated	Temozolomide	miR-25-3p confers temozolomide resistance though suppressing FBXW7, thus inducing c-MYC and cyclin E expression	miR-25-3p antagomir	(194)
miR-188-5p	BC	*In vitro*	Upregulated	Doxorubicin	miR-188-5p impedes response to apoptosis through the FBXW7/c-MYC axis	Honokiol; miR-188-5p antagomir	(195)

Furthermore, several therapeutic agents or signaling molecules are involved in the regulation of miRNAs and its downstream FBXW7, so that miRNAs or its upstream proteins can be critical targets to enhance the sensitivity of chemo-, immuno-, and radiotherapies. We find that inhibiting the upstream regulators of miRNAs can be of great value to combat therapeutic resistance. For example, serving as negative regulators of miR-223, the Notch and Akt inhibitors have shown efficacy in increasing drug sensitivity of T-ALL and NSCLC, respectively. Another example is that the bioactive natural product honokiol downregulates miR-188-5p and further attenuates the inhibition of the FBXW7/c-MYC axis, thus increasing doxorubicin sensitivity in BC cells (195). Nonetheless, the *in vivo* combined with *in vitro* studies on miRNA upstream regulators are relatively rare, the mechanisms of miRNAs targeting FBXW7 to confer therapeutic resistance of cancers remain elusive, and the effective delivery of miRNA mimics or anti-miRNAs to realize significant clinical values is awaiting to be elucidated.

### 3.2 Waking-up Therapy

CSCs, which consistently dwell within the tumor microenvironment, is defined as a subpopulation of cancer cells with the ability of self-renewing and differentiating into multiple types of heterogeneous cancers (196) like normal stem cells. The stemness properties maintain CSCs in a quiescent and non-proliferative state (also called dormancy), persistently staying in the G_0_ stage and infrequently entering the cell cycle, which is associated with therapeutic resistance, maintenance, as well as anti-apoptosis (197).

Serving as a suppressor protein, FBXW7 attenuates tumor stemness by orchestrating different pathways. EMT, a critical cellular process as mentioned before, is associated with acquisition of stem cell traits of normal as well as cancer cells (198, 199), and functions as a crucial regulator of CSCs (101, 200). The mTOR complex (composed of mTORC1 and mTORC2) has been reported to play a key role in activating the EMT process (201), which is one of FBXW7’s substrates. In colon cancer cells, FBXW7 deficiency results in accumulation of mTOR, thus in turn favoring EMT-related stem-like properties (202). The ZEB family includes ZEB1 and ZEB2, both of which are crucial transcription factors subordinated to EMT-inducing transcription factors (EMT-TFs), which are usually endowed with stemness features (203). ZEB1, serving as a transcription factor promoting the EMT process (204), is associated with the stem-like properties of cholangiocarcinoma (CCA) cells, which can be explained by the fact that FBXW7 dampens EMT and stemness of CCA cells *via* the mTOR/ZEB1 signaling pathway (205). ZEB2 can be directly bound and degraded by FBXW7, and the FBXW/ZEB2 axis modulates stemness/differentiation, therapeutic resistance, as well as metastasis in CRC cells (206).

Similar to the EMT process, the Wnt/β-catenin signaling exerts its pivotal role in regulating functions of normal stem cells as well as CSCs (207, 208). Cancer-associated fibroblasts (CAFs), the stromal cells dwelling in tumor microenvironment, is involved in tumor progression. CAFs can deliver exosomal miR-92a-3p to CRC cells, which in turn activates Wnt/β-catenin pathway by suppressing FBXW7 and Modulator of apoptosis 1 (MOAP1) and finally promotes cell stemness (184). Moreover, the exosomal miR-19b derived from CRC cells promotes stemness by inhibiting FBXW7 expression, which subsequently induces Wnt/β-catenin signaling pathway (193). In perihilar cholangiocarcinoma (pCCA), the high mobility group A1 (HMGA1) interacts with thyroid hormone receptor interactor 13 (TRIP13), and the HMGA1-TRIP13 axis promotes cell stemness by downregulating FBXW7 expression and thus stabilizing c-MYC, the latter in interplay with Wnt/β-catenin signaling (209).

Contrary to the aforementioned onco-suppressive role, FBXW7 is capable of promoting cancer progression by maintaining stemness of cancer cells. FBXW7 targets positive regulators of cell cycle, such as cyclin E (210) and c-MYC (211) for ubiquitination and subsequent degradation, hence inducing exit from cell cycle and maintaining cells within the quiescent state (212, 213). Among these substrates, c-MYC is pivotal to reverse the quiescent state of CSCs and thus induce them into cell cycle. For example, FBXW7 deficiency gives rise to premature loss of normal HSC, which largely boils down to c-Myc-triggered active cell cycling, showing that FBXW7 is significant for hematopoietic stem cell (HSC) maintenance (214). In chronic myeloid leukemia (CML), FBXW7 expression is integral to initiation, progression, and maintenance of leukemia stem cells (LSCs) associated with downregulation of c-MYC (215), and ablation of FBXW7 results in increased c-MYC and activated p53 pathway, further promoting LSC apoptosis (216). In addition to non-solid tumors, FBXW7 also exerts a functional role in CSCs of solid tumors. Under the condition of cycle arrest due to anticancer agent therapy, c-MYC expression is reduced due to the elevated level of FBXW7, and upregulated FBXW7 confers chemoresistance on colorectal CSCs (217). Depletion of FBXW7 dominantly reduces gefitinib-resistant CSC population in NSCLCs and alters cell cycling in the G_0_/G_1_ phase (218). Similarly, FBXW7 diminution enhances the proliferative identity of breast CSCs and the sensitivity to paclitaxel therapy *in vitro*, and FBXW7 ablation combined with chemotherapy exhibits longer survival period and more favorable prognosis of mice *in vivo* (219).

Based on this, waking up CSCs from a quiescent to a proliferative state by targeting its internal molecules and cues can be a potential strategy to break the vital bottleneck. Waking-up therapy has been applied for CML patients in several clinical trials, with no distinct risk detected in combination with standard regiments (219). Nonetheless, there remain several barriers to finally realize efficacy of the waking-up therapy targeting FBXW7. First, we wonder how to leverage the bi-functions of FBXW7 in stemness maintenance in order to countervail therapeutic resistance. Just as mentioned before, on the one hand, FBXW7 has been reported to dampen stemness properties of CSCs through the EMT and Wnt/β-catenin signaling pathways; on the other hand, the genetic silencing of FBXW7 confers accumulation of c-MYC, finally repressing the state of dormancy and sensitizing cancer cells to therapies, which has been substantiated *in vitro* and *in vivo* studies. Second, the delivery of drugs may bring out side effects on cancer cells aside from CSCs, such as alterations of proliferation, apoptosis, invasion, and metastasis based on our general concept of FBXW7 as a tumor suppressor. Third, although a part of these thorniest puzzles can be handled out by virtue of combined therapies, there is still a long way to go for real clinical applications on cancer patients not just mice models.

### 3.3 Therapies Based on Regulating Circadian Rhythms

Circadian rhythms can govern a large array of biological processes linked to body homeostasis, characterized by periodic oscillations of 24 h, which is modulated by an endogenous clock system (220, 221). The autonomous, organized circadian system is composed of three critical components including input pathways, central pacemaker, and output pathways, among which the central pacemaker is located in suprachiasmatic nucleus (SCN) of anterior hypothalamus (220). Circadian rhythms are orchestrated by intricate transcriptional–translational system feedback loops, which are governed by clock genes. Circadian locomotor output cycles kaput (CLOCK) and brain and muscle ARNT-like 1 (BMAL1) heterodimerize as a complex and bind to E-boxes of promoters on clock-controlled genes, which triggers their transcriptions containing Period (Per) and Cryptochrome (Cry) families during the day; proteins encoded by Per and Cry families suppress CLOCK/BMAL1 transcription activity during the night (222). In addition, core clock proteins are under the control of the orphan nuclear receptors REV-ERBs (including REV-ERBα and REV-ERBβ) and retinoic acid receptor-related orphan receptors (RORs), respectively (223). Dysregulation of circadian rhythms can contribute to metabolic disorders, carcinogenesis, immune inefficiency, poorer cancer prognosis, and attenuation of drug efficacy on cancer patients (221, 224).

The circadian clock protein cryptochrome 2 (CRY2) is overexpressed in specimen of CRC patients, correlated with chemoresistance and poor prognosis. Furthermore, FBXW7 negatively regulates CRY2 *via* a ubiquitin-dependent pathway and potentiates chemosensitivity of CRC cells, which proposes the method to enhance the antitumor efficacy targeting clock proteins (225). After being phosphorylated by cyclin-dependent kinase 1 (CDK1), the negative regulator of clock transcription REV-ERBα can be degraded by FBXW7, favoring amplitude of clock transcription. Meanwhile, rhythm disruption induced by FBXW7-specific deficiency of liver perturbs the circadian homeostasis of lipid and glucose metabolism (226), recognized as a critical impediment to tumorigenesis. In addition, as an integral part of regulating cell metabolism, mTOR signaling pathway is detected to show an oscillated 24-h rhythm in RCC cells in part caused by fluctuated expression of FBXW7 protein, whose pattern shows anti-phase of rhythm of mTOR protein. Experiment results indicate that D-site binding protein (DBP) binds to D-site element in the FBXW7 promoter and also exhibits a circadian rhythm of protein levels, thus regulating the transcriptional activity of FBXW7. The higher activity of mTOR in tumor-bearing mice is correlated to better efficacy of everolimus, underpinning the significant impacts of circadian rhythm of mTOR signaling on the antitumor therapies (82).

Based on the association of FBXW7 and circadian rhythms, we outline this potential therapy in the bud, which elevates therapeutic responses through regulation of circadian rhythms involving FBXW7. Dysregulated FBXW7 expression renders tumorigenesis by conferring disrupted circadian rhythms as well as metabolism, thus providing a potential target for curbing tumorigenesis and elevating therapeutic responses. In addition, FBXW7 modulates the activity of critical molecules residing in the circadian system (such as mTOR), and the activity of mTOR in tumor-bearing mice is correlated to better efficacy of mTOR inhibitor everolimus, so leveraging the oscillated therapeutic sensitivity is critical to our therapy. As a consequence, FBXW7 expression is upregulated to maintain circadian rhythm and metabolism homeostasis, and chronotherapy is provided when the activity of molecules within the circadian system regulated by FBXW7 is the highest, both of which can be taken advantage of in order to attain better efficacy. Nevertheless, the potential therapy we have proposed is in the bud, which urgently requires in-depth exploration of critical molecules within the clock system as well as their oscillated rhythms and fluctuated sensitivity at different times of the day.

## 4 Concluding Remarks

UPS has been heralded as the main degradation mechanism of eukaryotes, located at the crossroads of multiple cellular processes. Considering the relationship between abnormalities of UPS and carcinogenesis, we focus on FBXW7, a component of the SCF ubiquitin ligase complexes, which mainly serves as a tumor suppressor through ubiquitin-mediated degradation of its substrates, such as c-MYC, mTOR, MCL-1, Notch, c-Jun, and cyclin E. Being devoid of FBXW7 results in the accumulation of target proteins, demonstrated in *in vitro* and *in vivo* studies to impact the initiation, development, relapse, and therapeutic responses of multiple cancers. The expression levels of FBXW7 in most malignancies are downregulated compared to normal tissues, such as glioma, lung cancer, liver cancer, urothelial cancer, ovarian cancer, and melanoma (18), which have a positive correlation with prognosis of patients, aside from liver cancer and melanoma. Other studies have also shown that expression pattern alterations of FBXW7 are correlated with malignancy progression of cancer cells, as well as clinicopathological classification and prognosis of patients who are predisposed to relapse, so FBXW7 may figure as a predictor of initiation, development, and prognosis of cancers (213, 227, 228).

Here, we discuss how FBXW7 influences the hallmarks of cancer that are subdivided into 7 parts. We infer that the pathways underlying cancer hallmarks can be intricate and interwoven with each other, for example, that the crucial transcriptional factor c-MYC regulated by FBXW7 participates in proliferation, apoptosis, invasion and metastasis, energy reprogramming, and immune evasion of cancers. In light of c-MYC as one of the undruggable genomic drivers, attenuating oncogenic c-MYC-induced cellular processes by targeting FBXW7 can be new therapeutic targets, which have been elaborated in previous sections. FBXW7’s role as a tumor suppressor has been pervasively explored. In practice, FBXW7 figures as a double-edged sword impacting tumorigenesis, especially its role in maintenance of cancer cell stemness.

Tumor burden and growth kinetics, tumor microenvironment, cell death inhibition, and EMT contribute to therapeutic resistance (229), which can be leveraged by targeting FBXW7 to elevate therapeutic sensitivity and the prognosis of patients. We provide three prospective strategies to deal with this problem. First, we take advantage of miRNAs interacting with FBXW7 to regulate therapeutic sensitivity, among which miR-223 has gained great attention of many researchers. miR-223 is an evolutionarily conserved microRNA mainly expressed in hematopoietic cells, which comes from endogenous expression or releasing of exosomes and EVs. miR-223 has been detected to be upregulated in T-ALL, GC, NSCLC, and HCC *in vitro* and *in vivo*, mediating therapeutic resistance presumably through FBXW7-regulated proliferation, apoptosis, EMT, and autophagy. More *in vivo* studies should substantiate the exact roles of microRNAs in tumorigenesis, and the effective delivery of miRNA mimics or anti-miRNAs remains to be elucidated, emerging as a novel target therapy in cancers over the years (180). Second, given FBXW7’s role in the maintenance of CSCs, we introduce the waking-up therapy regulating cell cycle by targeting the switch between quiescent and proliferative states, and propose several potential risks for application of this therapy. Through the genetic ablation of FBXW7, LSCs are predisposed to enter the cell cycle and are sensitive to chemotherapy. FBXW7 diminution combined with other drug therapies demonstrates great efficacy in patients of CML chronic phase. Last but not least, we unravel the relationship between FBXW7 and circadian rhythms, the latter in close interaction with metabolism, immune functions, and carcinogenesis. In order to maximize the therapeutic efficacy, taking advantage of chronotherapy or targeting critical molecules of the circadian machinery emerges as a new treatment paradigm in cancers in the foreseeable future. In addition, we are looking forward to incorporating additional hallmarks, continuing to explore FBXW7’s role in other facets of cancer.

## Author Contributions

WS, QZ, CR, WL, and XJ conceived and designed the study and analyzed the results. Other authors performed analysis procedures. WS wrote the manuscript. QZ and WL revised the manuscript. All authors contributed to the article and approved the submitted version.

## Funding

This study was supported by the National Natural Science Foundation of China [Grant Nos. 81773179 and 81272972 (CR); Grant No. 81472355 (XJ)] and the Hunan Provincial Science and Technology Department [Grant No. 2016JC2049 (CR); Grant No. 2014FJ6006 (XJ)]. This work was also supported by the Students Innovations in Central South University of China (No. 2021105330188).

## Conflict of Interest

The authors declare that the research was conducted in the absence of any commercial or financial relationships that could be construed as a potential conflict of interest.

## Publisher’s Note

All claims expressed in this article are solely those of the authors and do not necessarily represent those of their affiliated organizations, or those of the publisher, the editors and the reviewers. Any product that may be evaluated in this article, or claim that may be made by its manufacturer, is not guaranteed or endorsed by the publisher.
